# Experiences and Views of African Australian Consumers on the Role of Spirituality, Religion and Culture on Mental Health

**DOI:** 10.1111/inm.70314

**Published:** 2026-07-17

**Authors:** Bekithemba Sibanda, Bindu. Joseph, Michael Olasoji

**Affiliations:** ^1^ Federation University, Australia Berwick VIC Australia

**Keywords:** Africans, Australian, culture, mental health, religion, spirituality

## Abstract

This study explored the lived experiences of African Australian consumers and their perspectives on the role of spirituality, religion and culture in shaping mental health and engagement with mental health services. A qualitative phenomenological design informed by transcultural nursing perspectives was used. Semi‐structured interviews were conducted with 15 African Australian participants aged 18–25 years from diverse African backgrounds, including Zimbabwe (*n* = 2), Ethiopia (*n* = 3), South Sudan (*n* = 5), Somalia (*n* = 4) and Ghana (*n* = 1). Interviews were audio‐recorded and transcribed verbatim. The data were analysed using Colaizzi's phenomenological method of analysis, which involved identifying significant statements, formulating meanings, clustering themes and developing an exhaustive description of participants' experiences and interpretations of mental health within cultural and spiritual contexts. Three interconnected themes were identified: living between two worlds, seeking healing beyond medication and breaking the silence. Participants described migration‐related stress, disrupted social networks and tensions between collectivist cultural expectations and individual identity as contributing to psychological distress. At the same time, spirituality, religious practices and culturally grounded community networks were identified as important coping resources that fostered resilience and social support. Participants reported mixed experiences with formal mental health services, noting that biomedical approaches often overlooked cultural and spiritual dimensions of wellbeing, while culturally responsive care enhanced trust and engagement. The findings highlight the importance of culturally and spiritually responsive mental health care to improve service engagement and support the wellbeing of African Australian communities.

## 1 Introduction

International migration has increased substantially worldwide, with approximately 281 million people living outside their country of origin (United Nations 2021; World Health Organization 2020a). Despite global commitments to equitable health care, migrants continue to experience disparities in access to quality mental health services compared with host populations (Renzaho et al. 2015; Fauk et al. 2021; Sibanda et al. 2026). However, little is known about how African Australian migrants experience mental health care, particularly how spirituality, religion and culture shape these experiences, an important gap given the need for culturally responsive mental health nursing practice and policy.

Australia is one of the most culturally diverse countries globally, with migration contributing to diversity in language, religion, cultural practices and country of origin (Hiruy and Mwanri 2014). Following immigration reforms that dismantled the ‘White Australia policy’, migration from African countries to Australia has steadily increased (Bastian 2012). The 2021 Australian census reported that more than 400 000 Australians identify as having African ancestry (Australian Bureau of Statistics 2022).

African Australian communities often maintain strong cultural traditions, spiritual beliefs and religious practices that influence understandings of health, illness and help‐seeking behaviours. In this study, spirituality refers to individuals' sense of meaning, connection or relationship with the sacred or transcendent; religion refers to organised systems of beliefs and practices associated with specific faith traditions; and culture refers to shared values, norms and practices that shape collective identity and social behaviour. While these constructs may overlap in practice, they are analytically distinguished in this study to better understand their influence on experiences of mental health care.

Understanding how these factors shape African Australian consumers' engagement with mental health services is important for mental health nurses and service providers working in increasingly multicultural settings. Culturally responsive mental health care requires greater insight into how consumers interpret mental health, illness and treatment within their social and spiritual contexts.

The aim of this study is to explore African Australian consumers' experiences and views of mental health care in Australia, with a particular focus on how spirituality, religion and culture shape these experiences. This study forms part of a broader phenomenological investigation examining the role of spirituality, religion and culture in the mental health of African Australians. This study is novel because it explores African Australian consumers' lived experiences of mental health care in Australia through a phenomenological lens that explicitly examines the intersecting roles of spirituality, religion and culture. Such insights may inform culturally responsive mental health nursing practice and contribute to improved service engagement among African Australian communities.

## 2 Background

Culture, spirituality and religion are widely recognised as important determinants of mental health and help‐seeking among people of African descent. Evidence from the United Kingdom, Canada, the United States and South Africa demonstrates the significant influence of these interconnected factors on mental health experiences and service engagement. In the United Kingdom, Black African service users were found to interpret mental illness and recovery predominantly through a religious framework, with faith providing a lens for understanding mental distress and supporting recovery (Tuffour 2020). Similarly, a Canadian study reported that, among people of African descent, spirituality is inseparable from identity and wellbeing, being deeply embedded within family relationships, cultural values and belief systems (Mbakogu and Richards 2026). In the United States, a study of Somali immigrants living in Minnesota found that participants' understandings, beliefs and experiences of mental health care were best interpreted within their cultural context (Wolf et al. 2016). Consistent with these international findings, a South African study demonstrated that religious and cultural beliefs are central to individuals' understanding of mental health and strongly influence help‐seeking behaviours (Mahomed 2023).

In Australia Mental health conditions are highly prevalent. According to the National Mental Health Workforce Strategy 2022–2032, one in five Australians aged 16 years and over (20%) experience a mental health condition in any given year, and almost half (45.5%) will experience a mental health condition at some point in their lifetime (Australian Government Department of Health 2022). Despite this substantial burden, national mental health policy highlights persistent gaps in data relating to the mental health of Culturally and Linguistically Diverse (CALD) communities and emphasises the need for culturally responsive practice across the mental health workforce (Australian Government Department of Health 2022). The Productivity Commission (2020) further identifies cultural capability as essential for addressing cultural and health literacy barriers that impede access, engagement and quality of care for CALD consumers.

At a state level, the Royal Commission into Victoria's Mental Health System (2021) concluded that the system is ‘fundamentally failing’ people from CALD backgrounds, underscoring the urgency of reform. International evidence similarly demonstrates disproportionate mental health challenges among migrant populations. For example, global studies report elevated rates of anxiety and post‐traumatic stress disorder among African migrants (James et al. 2022). In the Australian context, African‐born young people have been found to have up to 10 times the likelihood of experiencing a psychotic disorder compared with their Australian‐born peers (O'Donoghue et al. 2021).

Although spirituality, religion and cultural identity are well‐established determinants of mental health and wellbeing, research consistently shows that these dimensions remain under‐recognised in assessment, treatment planning and service delivery (Sibanda et al. 2025). Cultural beliefs shape how mental health symptoms are interpreted, when and how help is sought, and the acceptability of different treatment approaches (Molla et al. 2025). Incorporating spirituality and religion into mental health care has been shown to enhance outcomes by strengthening social support, community connection and resilience (Molla et al. 2025; Sibanda et al. 2025). However, there is limited empirical evidence capturing the lived experiences of African mental health consumers in Australia, particularly regarding how their cultural, spiritual and religious identities influence their engagement with mental health services.

This gap in evidence limits the development of culturally capable mental health services that respond to the needs of African communities in Australia.

### 2.1 Overall Aim

To explore the lived experiences of African mental health consumers in Australia, with a focus on how religion, spirituality and cultural identity shape their mental health journeys and interactions with the mental health system.

### 2.2 Specific Objectives

1. To examine how African consumers understand and interpret mental health and mental illness within their cultural, spiritual and religious frameworks.
2. To explore the experiences of African consumers in accessing and engaging with Australian mental health services.
3. To identify barriers and facilitators to culturally, spiritually and religiously responsive mental health care.
4. To generate insights that can inform culturally capable mental health practice and policy for African communities in Australia.

## 3 Methods

### 3.1 Theoretical Framework

This study examines the experiences and views of African Australian consumers on the role of spirituality, religion and culture in mental health and is guided by Leininger's Theory of Transcultural Nursing (Leininger 2002). The framework supports exploration of how cultural beliefs, values and lifeways shape health experiences, with attention to both cultural similarities and differences (McFarland and Wehbe‐Alamah 2019). It emphasises holistic, culturally congruent care as essential for meaningful and effective health promotion (Baldonado et al. 1998).

The framework guided the study's focus on consumers' cultural, spiritual and religious interpretations of mental health and informed the development of interview questions that explored culturally embedded meanings of mental illness, help‐seeking and coping. Data collection was shaped by Leininger's emphasis on culturally congruent engagement, with interviews conducted in a flexible, respectful manner that allowed participants to express their cultural and spiritual worldviews in their own terms. During analysis, the framework provided a lens for interpreting how participants' narratives reflected culturally shaped understandings of mental health, enabling a systematic examination of how cultural values, spiritual beliefs and religious practices influenced their responses to mental illness and preferred support strategies.

### 3.2 Study Design and Approach

This study employed a qualitative descriptive phenomenological approach to explore the lived experiences and perceptions of African Australian consumers regarding the influence of spirituality, religion and culture on mental health. Descriptive phenomenology seeks to describe and understand the essence of individuals' lived experiences as they are perceived and interpreted by participants while minimising researchers' preconceptions (Peoples 2020). This approach was considered appropriate because it enabled an in‐depth exploration of how participants understood and attributed meaning to spirituality, religion and culture within the context of their mental health experiences.

The study was informed by Colaizzi's descriptive phenomenological methodology, which provides a rigorous and systematic framework for exploring and describing lived experiences. Colaizzi's approach is well suited to research seeking to understand the meaning participants assign to a particular phenomenon while ensuring that interpretations remain grounded in their accounts. This methodological framework supported the generation of rich, detailed descriptions of participants' experiences and enhanced the credibility and rigour of the study. The procedures used for data analysis are described in Section 3.4.

### 3.3 Participants and Data Collection

Participants were recruited using purposive convenience sampling combined with a snowballing technique to obtain a diverse range of perspectives (*n* = 15). Snowball sampling is particularly useful when recruiting participants from marginalised populations (Parker et al. 2019). Initial participants were identified through study flyers emailed to Directors of Nursing and Case Managers within mental health services, who acted as gatekeepers by sharing a study flyer containing general information and researcher contact details and subsequently distributed the information to mental health nurses and Case managers shared the study invitation with eligible consumers; however, they did not participate in the recruitment decision or have access to participants' responses. Interested individuals contacted the research team directly via email or phone call. Following their interviews, participants were invited to refer other potentially eligible individuals within their networks, consistent with the snowball sampling approach.

The inclusion criteria were as follows: (1) self‐identification as African Australian, (2) aged 18 years or older, (3) living in Australia, and (4) having a self‐reported diagnosis of a mental health condition. Individuals were excluded if they were under 18 years of age, unable to provide informed consent or unable to participate in an interview conducted in English.

Data were collected through semi‐structured, in‐depth interviews with 15 African Australian participants. Interviews were conducted either face‐to‐face or via secure online videoconferencing, depending on participant preference and location, and followed a semi‐structured interview guide. This approach enabled participants to discuss their experiences in depth while allowing flexibility to explore emerging ideas (Adeoye‐Olatunde et al. 2021). Example interview questions included: ‘In your view, what is the role of spirituality and culture in your personal recovery?’, ‘Can you describe your experiences of mental health care in Australia?’, ‘How do spirituality or religion influence your understanding of mental health?’, and ‘How do cultural beliefs shape the way you seek support for mental health concerns?’. The study was carried out by a team of three researchers from culturally and linguistically diverse (CALD) backgrounds, who were responsible for both conducting and analysing the interviews. While the researchers' own cultural backgrounds did not always align directly with those of the participants, their varied cultural and linguistic viewpoints enriched the reflexive conversations held during data collection and analysis. This diversity helped ensure that the experiences of African Australian participants were interpreted in ways that were culturally attentive and responsive.

### 3.4 Data Analysis

Audio‐recorded interviews were transcribed verbatim using AVID Note transcription software. Each transcript was subsequently reviewed against the original recording to ensure accuracy. Selected quotations were retained to illustrate participants' accounts within the findings.

Data were analysed using Colaizzi's seven‐step descriptive phenomenological method, which is congruent with the descriptive phenomenological design adopted in this study (Table 1). Transcripts were read repeatedly to achieve immersion in the data and to develop a holistic understanding of participants' lived experiences. Throughout this process, the researchers bracketed their preconceptions and prior assumptions to support phenomenological reduction and enhance awareness of the data as experienced by participants.

**TABLE 1:** Summary of Colaizzi's seven step analysis and rigour strategies.

Colaizzi step	Description of activities	Rigour/trustworthiness strategies
Step 1: Familiarisation	Researchers read transcripts multiple times to gain a comprehensive understanding of participants' experiences.	Prolonged engagement with transcripts; verification of transcripts against audio recordings; use of field notes.
Step 2: Extracting Significant Statements	Statements directly related to spirituality, culture and mental health were identified across transcripts.	Collaborative identification by three researchers; maintaining an audit trail of analytic decisions.
Step 3: Formulating Meanings	Significant statements were interpreted to develop meanings grounded in participants' intended perspectives.	Reflexive note‐keeping minimising bias; team discussions to ensure interpretive consistency.
Step 4: Clustering Themes	Formulated meanings were grouped into theme clusters representing shared experiential patterns.	Iterative team review; comparison across transcripts; ensuring themes remained grounded in data.
Step 5: Developing an Exhaustive Description	All themes were integrated into a comprehensive description capturing the depth of the phenomenon.	Transparent documentation linking themes to supporting quotations.
Step 6: Identifying the Fundamental Structure	The exhaustive description was distilled into a concise statement representing the essence of carers' experiences.	Team validation of conceptual clarity and alignment with original data.
Step 7: Member Checking	Participants reviewed the preliminary findings to confirm the accuracy of interpretations.	Participant feedback used to validate authenticity and relevance of findings.
Ongoing Across All Steps	Reflexive practice, analytical dialogue among researchers, and systematic documentation.	Reflexivity, collaborative analysis, audit trail, and verbatim quotations used to enhance credibility, dependability and confirmability.

Significant statements pertaining to spirituality, religion, culture and mental health were systematically identified from each transcript. Formulated meanings were then derived from these statements, remaining grounded in participants' original accounts. These formulated meanings were subsequently organised into clusters of related ideas that reflected shared patterns across participants' experiences. Each cluster was reviewed iteratively against the original transcripts to verify that it accurately represented participants' accounts and maintained internal consistency.

The clustered meanings were then synthesised into an exhaustive description of the phenomenon, which was condensed into a concise statement capturing the fundamental structure of participants' shared lived experiences. Where feasible, participants were invited to review the findings to confirm that the interpretations accurately reflected their experiences.

All three researchers contributed to the analytic process. Throughout analysis, the team engaged in reflexive discussions to examine how their cultural backgrounds, professional experiences and assumptions may have influenced interpretation. Consensus on formulated meanings, thematic clusters, and the final thematic structure was reached through iterative dialogue, thereby enhancing the credibility and dependability of the analysis. The Transcultural Nursing framework was retained as an interpretive lens to support contextual understanding of participants' narratives, particularly in relation to how cultural values, spiritual beliefs and religious practices shaped their experiences of mental health and help‐seeking preferences. This framework informed the interpretation of the findings rather than guiding the analytic process itself.

## 4 Ethical Considerations

Ethical approval for this study was obtained from the Human Research Ethics Committee at Federation University Australia (Approval No. 2023/116). Participants were informed that their participation was voluntary and that they could withdraw from the study at any time without consequence; however, no participants chose to withdraw. Confidentiality and anonymity were maintained by assigning codes to participants and de‐identifying all audio recordings and interview transcripts. Participants were also assured that their responses would remain confidential and would not be linked to their identities.

## 5 Results

Fifteen participants were included in the study, representing diverse African backgrounds (see Table 2 for full demographic details).

**TABLE 2:** Participant demographics.

African Australian consumer	
Characteristics	No = 15 (7 females and 8 males)
**Age**	
≤ 20	5
21–30	4
31–40	2
41–50	3
> 50	1
**Country of origin**	
Somalia	4
South Sudan	5
Ethiopia	3
Zimbabwe	2
Ghana	1

Three interconnected themes were identified, describing how African Australian participants navigate mental health within the intersecting contexts of migration, cultural identity and Western mental health systems (Living between two worlds, Seeking Healing Beyond Medication and breaking the silence). These themes capture the tensions between cultural expectations, spiritual understandings of wellbeing and biomedical models of care.

### 5.1 Theme 1: Living Between Two Worlds—Acculturation, Cultural Expectations and Emotional Distress

This theme explores how the process of acculturation influences the mental health and personal relationships of African migrants. Participants' narratives reveal the emotional distress caused by navigating new cultural expectations, the protective role of cultural community ties, and the emotional burdens stemming from cultural and familial expectations.

#### 5.1.1 Cultural Transition as a Trigger for Psychological Distress

Participants consistently described heightened emotional distress linked to unfamiliar cultural demands and stigma surrounding mental health following migration. Many participants connected their emotional challenges to the tension between culturally embedded expectations and new social norms encountered in Australian society. For example, several participants reflected on the psychological impact of acculturation, with one noting,

I think that a lot more Africans get mental health issues when they come here… because of a myriad of acculturation factors (C11).

The transition to Australian society often involved navigating unfamiliar social norms, communication styles and approaches to mental health, which, for some participants, intensified feelings of isolation or frustration. Several participants described challenges in engaging with formal support services when cultural understanding was lacking. This was evident in accounts of disengagement from therapy due to perceived cultural differences,

I don't think therapy is a bad thing for any issues at all. I just think there was a cultural difference and her not understanding my circumstances and where my headspace was at, it just did not really mesh well or sit well with me. So, I stopped going after that one time (C7).

A few male participants reported that cultural norms, particularly those around masculinity, frequently stigmatise emotional vulnerability, making it difficult for men to openly acknowledge mental health struggles as explained by another participant,

Culture tells you… as a man, you can't be depressed… they don't recognise it. (C1).

#### 5.1.2 Loss of Community and the Loneliness of Individualistic Society

The loss of formal community supports and the transition to a more individualistic society also emerged as common sources of distress. Participants contrasted the informal social support they experienced in their countries of origin with feelings of isolation in Australia; this is illustrated in the following account,

…I miss the informal structures back home; I miss turning up unannounced at my neighbour's doorstep…. Culturally you don't have to make an appointment to visit someone… back home I was never isolated and never felt lonely (C15).

Several participants reflected on how the absence of spontaneous, communal interactions can intensify loneliness and exacerbate mental health challenges. In contrast, participants highlighted the protective role of cultural events, viewing them as informal spaces for emotional support and social connection,

The amount of people that actually look forward to a cultural event and come to it. People of the same culture. To learn their dance. And also, not that. Meet up with their person… talk just to link up… you feel better by just attending the event (C9).

#### 5.1.3 Cultural Obligations and Family Expectations as Emotional Pressure

Family and cultural obligations further contributed to emotional tension, particularly when personal aspirations conflicted with traditional expectations around marriage and intimate relationships. Participants described the stress of navigating these obligations,

Mental illness for me was caused by stress, my parents took me back to… for a holiday, then married me to this stranger and we came back to Australia… we did not even know each other, we were strangers who had children together. I felt trapped as I did not want to disrespect my family. (C13).

Another participant described the ongoing pressure to meet parental expectations while protecting personal well‐being,

…And that adds extra pressure where you'd feel you're going against their wishes, culturally that is considered disrespectful. And then now you feel like you're letting them down because not to forget you want to make them happy knowing that they left their country. (C6).

### 5.2 Theme 2: Seeking Healing Beyond Medication—Faith, Culture and the Need for Holistic Care

This theme comprises three subthemes: medication as a ‘quick fix’ disconnected from cultural experience; spiritual practices as sources of resilience and healing; and the need for culturally responsive care. Many participants expressed dissatisfaction with biomedical approaches that relied primarily on medication, emphasising that effective healing extended beyond clinical treatments to include spiritual practices, cultural traditions and community connections, elements often absent from mainstream services.

#### 5.2.1 Medication as a ‘Quick Fix’ Disconnected From Cultural Experience

Most participants described medication as a ‘quick fix’ that was disconnected from their lived experiences and cultural contexts. Several expressed concern that biomedical approaches prioritised pharmacological treatment without adequately considering cultural, spiritual or emotional needs. As reflected in one of the accounts,

They rush into giving you medications… they don't consider my cultural background or religion, is there anything that anyone thinks could be done better instead of just giving you medications? There should be at least counselling. Yeah? (C8).

Concerns about medication side effects were also frequently raised, particularly in relation to their impact on daily functioning, social engagement and spiritual practices. For some participants, side effects disrupted meaningful aspects of life, including participation in religious activities,

Sometimes side effects outweigh the benefits… I stopped going to church as a result (C4).

This perception of urgency to medicate, without addressing underlying or culturally relevant issues, was commonly criticised across accounts. Participants emphasised the need for more holistic and culturally responsive approaches to care, including being asked about their beliefs and personal context,

So the remedy, they rush the medication onto people… try this type of system rather than rushing people into medication because… I need to be asked about my beliefs too. (C7)

For many, heavy medication led to feelings of disconnection, not only from themselves but also from their community and cultural identity. The side effects sometimes made individuals feel ‘like a zombie’, distancing them from the social networks and communal life essential in African cultural contexts,

Medication is too much, they need to reduce it… you're always gonna have medication and in the end, you end up looking like a zombie. In my community, we have people around us all the time and they visit me in numbers, but if I am a zombie I can't communicate with people. I feel lonely and isolated, and that makes me worse… (C13).

I know some people that have just turned into zombies because of medication… you start to lose people and friends through it… and culturally we struggle without family and friends. (C1).

Concerns about medication changing personality and daily functioning were particularly poignant, as maintaining cultural identity and personal agency are deeply valued,

But sometimes side effects outweigh the benefits in some cases. And that's why a lot of us don't want to be on medication anymore or we stop using it and like that because it does change us as people…. I stopped going to church as a result of side effects (C4).

Some participants viewed medication only approaches as superficial, emphasising the need for treatment that considers their cultural experiences and holistic well‐being,

Just giving people medication is… that's kind of like a band‐aid solution because you're just going to go straight back into it… include their cultural and spiritual beliefs too… their cultural food, prayer, and family (C2).

#### 5.2.2 Spiritual Practices as Sources of Resilience and Healing

Faith and cultural practices emerged as important sources of resilience and emotional grounding. Engagement in spiritual practices was linked to comfort, identity affirmation and a sense of healing. Several participants shared examples such as,

Like I said, when I started reading my own Bible for my own spiritual health… that is where my spiritual affirmations came from… affirmations became my source of healing (C11).

Similarly, all Muslim participants spoke about the spiritual and emotional benefits of religious practices, underscoring how spiritual spaces could offer peace and a sense of grounding. One participant shared how attending the mosque and reading the Quran supported their well‐being,

So I'm Muslim… I find I feel well when I attend the mosque and read the Quran (C10).

In addition to religious faith, some participants turned to traditional healing practices passed down through generations. These methods were deeply embedded in cultural identity and were often seen as a way of reconnecting with one's roots,

My family have suggested that I go back home to engage in traditional healing practices including Sangoma (traditional healer) (C14).

…Staff did not allow me to leave the ward… I wanted to go back home, back in …but I was told I was on a treatment order… (C13).

#### 5.2.3 The Need for Culturally Responsive Care

Several participants highlighted the perceived importance of culturally responsive care. Participants valued clinicians who demonstrated understanding of their cultural context, as well as spiritual or community leaders who could provide guidance consistent with cultural values,

To be honest, … it will be much better if we had more African nurses…, I don't see a lot of African nurses. Having more African nurses and doctors will help a lot. (C10).

Other participants described the emotional distance and miscommunication that can occur when clinicians do not share or attempt to understand their cultural context. Furthermore, emphasised the profound sense of disconnect they experienced,

They don't understand me. They don't understand the struggles I'm going through or what another person is going through because there's already that initial disconnect, and no matter how much inclusivity, you need it from (C6).

Many participants who had access to culturally aligned clinicians reported feeling more supported and understood. Participant also shared their relief in finally receiving care from someone who resonated with their background,

I am happy now; I have a case manager who understands me. We call him Habesha (pan‐ethnic identifier). In the past, I had someone who did not understand me (C14).

Additionally, a few participants viewed culturally responsive care as extending beyond health professionals to include spiritual and community leaders. Participants highlighted the importance of involving familiar and trusted figures in their mental health assessment,

I needed my pastor and a family member to be involved in my assessment… That way they would have understood that I was not mad. (C4).

### 5.3 Theme 3: Breaking the Silence—Stigma, Knowledge Gaps and Community Pathways to Support

This theme explores how stigma, limited mental health awareness and cultural perceptions shape help‐seeking within African Australian communities. It encompasses three interconnected subthemes: mental health as a hidden or misunderstood issue; education as a pathway to reducing stigma; as well as community, faith and culturally competent care as pathways to support.

#### 5.3.1 Mental Health as a Hidden or Misunderstood Issue

Participants also highlighted culturally competent healthcare as critical for fostering trust and improving engagement with mental health services. A few participants consistently described mental health as a culturally sensitive issue, often hidden or misunderstood within African Australian communities. Stigma and limited awareness were reported as barriers to help‐seeking, particularly among women and new mothers,

A greater majority of… ladies that I speak to, they went through postnatal depression and a lot of them didn't even realise that was a thing… depression does not exist in my culture… (C10).

Another participant added,

Africans like to deal with things themselves first, …Some think oh it's this one time. But that one time can just take you off the edge (C7).

Cultural norms, particularly around masculinity, contributed to stigma by discouraging vulnerability and help‐seeking,

You're gonna be scared to be looked at by the community as a weak person… So, it deters you from seeking help (C6).

#### 5.3.2 Education as a Pathway to Reducing Stigma

Education was identified as a critical strategy for reducing stigma and was closely linked to broader community‐based approaches to mental health support (Section 5.3.3). A greater majority of participants emphasised that culturally tailored awareness programs could help normalise conversations about mental health within African communities, thereby creating a foundation for engagement with both community and formal support systems.

Several participants described stigma as deeply embedded and reinforced by silence around mental illness. For example, one participant highlighted the taboo nature of the topic, stating:

Mental illness is taboo… removing stigma… we have to destigmatise mental illness (C11).

Similarly, another participant emphasised the role of cultural normalisation, noting:

If we started as Africans normalising that… the stigma and shame… would not be there (C9).

#### 5.3.3 Community, Faith and Culturally Competent Care as Pathways to Support

Building on the role of education in reducing stigma, a few participants highlighted the importance of trusted community structures and culturally competent healthcare in improving mental health awareness and engagement with services. Faith and community leaders were described as influential figures who could operationalise educational efforts by embedding mental health knowledge within familiar and trusted settings.

Participants suggested that equipping religious leaders with mental health knowledge could facilitate culturally appropriate conversations and encourage help‐seeking within community contexts,

It will be good for a few church leaders to be trained and then they teach the congregation about mental health (C5).

In addition to community‐based approaches, participants emphasised the importance of culturally competent healthcare providers as a complementary pathway to improving engagement with formal services. This was closely linked to the broader role of education in addressing stigma, as increasing cultural understanding among healthcare professionals was seen as essential for building trust and fostering more inclusive care environments.

Several participants highlighted that understanding cultural values, beliefs and lived experiences is critical to reducing barriers to help‐seeking. For example, one participant suggested that enhancing cultural awareness among healthcare professionals could serve as a practical starting point, stating:

I think Training nurses about different cultures will be a good starting point (C1).

## 6 Discussion

This study explored how African Australian consumers experience and interpret the roles of culture, spirituality and religion in their mental health. Analysis identified three interconnected themes: living between two worlds, seeking healing beyond medication and breaking the silence. Viewed through the lens of Madeleine Leininger's Transcultural Nursing theory, the findings illustrate how culturally embedded beliefs, communal practices and spiritual worldviews shape mental health experiences, coping strategies and engagement with healthcare services.

A central finding of the study was the significant impact of acculturation stress on participants' mental wellbeing. Participants described experiences of isolation, disrupted social networks and tensions between traditional cultural expectations and individual identity following migration. Cultural obligations, including expectations related to family roles and arranged marriages, were described as emotionally burdensome when they conflicted with personal aspirations. These findings align with both local and international research demonstrating that migration‐related stress and social dislocation can increase the risk of depression and psychotic disorders among African migrant populations (O'Donoghue et al. 2021; James et al. 2022; Tortelli et al. 2015).

Participants also emphasised the psychological impact of losing culturally familiar communal structures following migration. Informal social interactions, extended family support and participation in cultural gatherings were described as central to wellbeing in participants' countries of origin. Their absence in the Australian context often intensified loneliness and emotional distress. These findings reinforce evidence that culturally meaningful social networks can buffer the psychological impact of acculturation stress and support wellbeing among African migrant communities (Abur and Mphande 2020).

The study further revealed complex experiences in navigating formal mental health treatment. Participants reported that biomedical approaches, particularly medication‐focused interventions, did not always address the cultural and spiritual dimensions of their mental health needs. Some described negative medication side effects that limited their ability to engage in everyday social and cultural activities. These perceptions are consistent with previous research reporting higher levels of treatment dissatisfaction and non‐concordance with antipsychotic medications among Black patients in Western healthcare contexts (McQuaid and Landier 2018; Richards‐Belle et al. 2025). In contrast, spirituality, religiosity and traditional healing practices were consistently described as important coping resources (Tannerah et al. 2026). Participants highlighted the role of prayer, engagement with sacred texts such as the Bible or Quran, participation in faith communities, and consultation with traditional healers as integral strategies for managing emotional distress. These practices not only provided psychological comfort but also reinforced cultural identity and social belonging. Similar findings have been reported in studies demonstrating the protective role of spiritual and traditional healing practices in supporting mental wellbeing among African populations (Ikafa et al. 2022; Burns and Tomita 2015).

Cultural alignment in healthcare interactions was also identified as an important factor influencing trust and engagement with mental health services. Participants reported greater trust and openness when clinicians demonstrated cultural understanding or shared similar cultural backgrounds. This finding supports the principles of Transcultural Nursing, which emphasise that culturally congruent care strengthens therapeutic relationships and improves health outcomes. It also reflects broader evidence indicating that culturally responsive healthcare can enhance patient engagement and satisfaction among culturally diverse populations.

Barriers to mental health help‐seeking were also evident in participants' accounts. Limited mental health literacy, stigma surrounding mental illness and gendered expectations within communities contributed to delays in recognising symptoms and accessing formal care. These findings are consistent with previous studies documenting stigma and delayed help‐seeking behaviours among African migrant populations (Tannerah et al. 2026; Conner et al. 2010; Meribe et al. 2025). Despite these barriers, participants identified several potential pathways for improving engagement with mental health services. Community education initiatives, collaboration with faith leaders and culturally tailored outreach were viewed as important strategies for improving awareness and reducing stigma (Tannerah et al. 2024). Training religious leaders to recognise signs of mental distress and facilitating collaboration between faith institutions and healthcare services were identified as promising approaches for bridging gaps between formal mental health systems and culturally grounded support networks. This aligns with research highlighting the importance of faith‐based social capital in promoting mental health awareness and support within African communities (Agyekum and Newbold 2016; Ali et al. 2022).

Overall, the findings demonstrate that African Australian mental health experiences are shaped by dynamic interactions between migration‐related stressors, cultural norms and spiritual resources. Acculturation stress and disrupted community ties function as key risk factors, while spirituality, religiosity, culturally aligned care and community engagement operate as protective mechanisms. These influences intersect to shape coping strategies, help‐seeking behaviours and mental health outcomes. Importantly, the findings extend the application of Leininger's Transcultural Nursing theory by demonstrating how cultural and spiritual resources can be actively integrated into mental health care for African Australian communities. Recognising and working with patients' cultural values, belief systems and communal support structures may strengthen therapeutic relationships, improve engagement with services and enhance resilience among culturally diverse populations.

## 7 Study Strengths and Limitations

As with most research, this study has both strengths and limitations. One key limitation is the relatively small sample size, which may introduce potential bias and limit the generalisability of the findings. Additionally, the study uses the term ‘African Australian’ to refer broadly to individuals of Sub‐Saharan African descent, despite the cultural diversity and differing values within this group. While this could be seen as a limitation, existing evidence suggests there are shared cultural characteristics that justify this grouping (Nzimakwe 2014).

A notable strength of the study lies in the cultural competence of the research team, which includes Sub‐Saharan African researchers and a researcher from a culturally and linguistically diverse (CALD) background (Tembo et al. 2025). This insider perspective enhances the depth and authenticity of the findings. Moreover, the involvement of experienced healthcare professionals, particularly those from CALD backgrounds, enriches the data and adds valuable insight into the complexities of culturally responsive mental health care.

## 8 Conclusion

This study highlights the complex ways in which culture, spirituality, religion and migration experiences shape the mental health of African Australian consumers. Guided by Madeleine Leininger's Transcultural Nursing theory, the findings demonstrate that mental health experiences are embedded within culturally and spiritually grounded belief systems that influence how distress is understood, managed and treated. Acculturation stress disrupted social networks, and tensions between collectivist expectations and individual identity emerged as key risk factors for psychological distress.

At the same time, spirituality, religiosity and culturally grounded community networks functioned as important protective resources that foster resilience, emotional regulation and social support. These findings reinforce the central premise of transcultural nursing that culturally congruent care improves engagement and wellbeing. Mental health services that acknowledge patients' spiritual beliefs, cultural values and communal support systems are better positioned to build trust and enhance care effectiveness.

Overall, integrating culturally and spiritually responsive approaches within mental health services may reduce barriers to care, strengthen engagement and support the wellbeing of African Australian communities.

## 9 Relevance to Clinical Practice

The findings of this study highlight the importance of culturally and spiritually responsive mental health care for African Australian consumers. Mental health nurses and other clinicians should acknowledge that culture, spirituality, religion and migration experiences shape how individuals understand psychological distress, seek help and engage with treatment. Providing culturally congruent care requires sensitive assessment of cultural and spiritual beliefs, as well as an acknowledgement of migration‐related stressors such as acculturation, disrupted social networks, stigma and limited mental health literacy. Where appropriate, clinicians should also collaborate with families, community organisations and faith leaders, in accordance with consumer preferences.

The findings further suggest that mental health services should incorporate models of care that align with cultural and spiritual values, recognising the role of communal and faith‐based support networks alongside evidence‐based clinical interventions. Community‐ and faith‐based mental health education may also help reduce stigma, improve mental health literacy and promote the earlier recognition of mental health challenges and help‐seeking. Services should additionally acknowledge the diverse experiences of African Australians, including the influence of collectivist values and the emergence of hybrid cultural identities among younger generations navigating cultural adaptation.

These findings also extend Leininger's Transcultural Nursing Theory by demonstrating how cultural identity, spirituality and community resources can function as protective assets that support mental health and resilience within migrant communities. Future research should evaluate culturally adapted interventions that systematically integrate these cultural and spiritual assets into mental health care and examine their effects on service engagement, treatment adherence and mental health outcomes among African Australian communities.

## Funding

Bekithemba Sibanda is supported by the Australian Government Research Training Program (RTP) Stipend and RTP Fee‐Offset Scholarship through Federation University Australia.

## Ethics Statement

This study received ethical approval from the Federation University Research Ethics committee (Approval No. 2023/116).

## Conflicts of Interest

The authors declare no conflicts of interest.

## Data Availability

The data that support the findings of this study are available from the corresponding author upon reasonable request.
